# Pharmacological Strategies for Manipulating Plant Ca^2+^ Signalling

**DOI:** 10.3390/ijms19051506

**Published:** 2018-05-18

**Authors:** Kjell De Vriese, Alex Costa, Tom Beeckman, Steffen Vanneste

**Affiliations:** 1 Department of Plant Biotechnology and Bioinformatics, Ghent University, Technologiepark 927, 9052 Ghent, Belgium; kjvri@psb.vib-ugent.be (K.D.V.); tobee@psb.vib-ugent.be (T.B.); 2 VIB Center for Plant Systems Biology, VIB, Technologiepark 927, 9052 Ghent, Belgium; 3 Department of Biosciences, University of Milan, 20133 Milan, Italy; alex.costa@unimi.it; 4 Instititute of Biophysics, Consiglio Nazionale delle Ricerche, 20133 Milan, Italy; 5 Lab of Plant Growth Analysis, Ghent University Global Campus, Songdomunhwa-Ro, 119, Yeonsu-gu, Incheon 21985, Korea

**Keywords:** calcium, Ca^2+^ channel, Ca^2+^ ATPase, calmodulin, Ca^2+^ chelator, Ca^2+^ ionophore

## Abstract

Calcium is one of the most pleiotropic second messengers in all living organisms. However, signalling specificity is encoded via spatio-temporally regulated signatures that act with surgical precision to elicit highly specific cellular responses. How this is brought about remains a big challenge in the plant field, in part due to a lack of specific tools to manipulate/interrogate the plant Ca^2+^ toolkit. In many cases, researchers resort to tools that were optimized in animal cells. However, the obviously large evolutionary distance between plants and animals implies that there is a good chance observed effects may not be specific to the intended plant target. Here, we provide an overview of pharmacological strategies that are commonly used to activate or inhibit plant Ca^2+^ signalling. We focus on highlighting modes of action where possible, and warn for potential pitfalls. Together, this review aims at guiding plant researchers through the Ca^2+^ pharmacology swamp.

## 1. Introduction

The ion Ca^2+^ is one of the most versatile signals in living organisms. In its most simple form, the sensing of Ca^2+^ elevation signals a breach of membrane integrity. This is true for the simplest Prokaryotes and the most complex, multicellular Eukaryotes. This basic signalling function is a by-product of the cell’s need to avoid high intracellular Ca^2+^ levels, as this can interfere with the primary metabolism due to the propensity of Ca^2+^ to form insoluble precipitates with phosphates. While still being an unwanted guest in high concentrations, evolution has integrated Ca^2+^ signalling into complex signalling cascades that fine-tune many, if not all cellular processes.

In Arabidopsis, more than 250 genes encode for proteins that have a predicted Ca^2+^ binding motif [1]. In addition, Ca^2+^ can modify protein-lipid interactions through direct electrostatic interaction with charges embedded in membranes [2]. This multitude of effects of Ca^2+^ in each cell implies rigid control mechanisms. Typically, cytosolic resting Ca^2+^ concentrations are kept in the submicromolar range, while the Ca^2+^ concentration in the vacuole and apoplast is in the millimolar range (reviewed in [3]) and submillimolar (50–500 µM) in the endoplasmic reticulum [3,4]. In the peroxisome (2 µM; [5,6]), mitochondrial matrix (200 nM; [7,8]), chloroplast (150 nM; [9]) and plastidic stroma (80 nM; [9,10]), Ca^2+^ concentrations are in the same range as the cytoplasm. Such steep concentration gradients allow generating a significant Ca^2+^ signal in immediate vicinity of the mouth of an activated Ca^2+^ channel. In combination with a slow Ca^2+^ movement in the cytoplasm (Ca^2+^ movement in Eukaryotic cells is about 10–25 µm/s; [11,12]), activation of a specific Ca^2+^ channel generates a local, discrete Ca^2+^ signal that can be interpreted by the Ca^2+^ sensing machinery in that volume of the cell. Immediately after entering the cytoplasm, Ca^2+^ channel activity is attenuated while Ca^2+^ efflux is activated, jointly effecting Ca^2+^ signal dissipation.

In plants, Ca^2+^ is a fundamental signal that is directly connected to plant developmental processes, such as tip growth of pollen tubes [13] and root hairs [14,15], and responses to phytohormones, biotic and abiotic stress such as salt, drought, heat, cold, touch, oxidative stress, and osmotic stress, to biotic stresses such as pathogen elicitors and nodulation factors, and phytohormones such as auxin, abscisic acid (ABA), gibberellic acid, and cytokinin (reviewed in [16,17,18]).

It is well known that distinct signals elicit very specific intracellular Ca^2+^ dynamics, so-called Ca^2+^ signatures that can often be described by their amplitude, duration, and frequency. A recent model for decoding Ca^2+^ signatures proposes that amplitude and duration of the Ca^2+^ signal selectively activates downstream targets which may include specific enzymes or signal transducers with different affinities for Ca^2+^ and Ca^2+^ binding kinetics [19,20]. The repertoire of the possible Ca^2+^ signatures is further expanded by spatio-temporal coordination of different Ca^2+^ channels within the same membrane or in membranes of different subcellular compartments. Together, these evolutionary adaptations have turned this simple bivalent cation into a very versatile messenger for a diverse set of signals. 

To understand the physiological relevance of Ca^2+^ signalling in plants, the best approach is via mutant analysis. However, as plant genomes often have undergone partial or even full genome duplications, the plant toolkit consists of mainly of large families of functionally redundant genes. This is illustrated with the poplar genome that encodes for 61 glutamate receptors (GLRs) [21]. This complication of the genetic analyses of gene function calls for selective pharmacology to interrogate the contribution of specific Ca^2+^ signalling modules. However, due to the large evolutionary distances between plants and metazoans [22], one cannot always modulate plant Ca^2+^ signalling with Ca^2+^ pharmacology that was developed in metazoan systems. In this review, we will provide an overview of commonly used strategies to dissect plant Ca^2+^ signalling, and highlight some of the pitfalls that are associated with them (Table 1).

## 2. Ca^2+^ Entry Mechanisms

Influx of Ca^2+^ ions via Ca^2+^-permeable channels is driven by transmembrane Ca^2+^ gradients, allowing for a passive influx of Ca^2+^ from the apoplast or internal Ca^2+^ stores (e.g., ER and vacuole) into the cytosol. Biophysical studies have shown that plant Ca^2+^ channels can be categorized into voltage-activated and voltage-independent Ca^2+^ channels (VICCs). Voltage-activated Ca^2+^ channels can be further subdivided into depolarization-activated Ca^2+^-permeable channels (DACCs) and hyperpolarization-activated Ca^2+^-permeable channels (HACCs) [143]. HACCs are primarily involved in guard cell closure and polar growth, and enable influx of Ca^2+^ in response to ABA, blue light and some elicitors [55,144,145]. While the regulatory mechanisms of these Ca^2+^ channels are not yet completely understood, several regulators are known to affect their activity, such as reactive oxygen species (ROS), phosphorylation, cAMP and cGMP, actin and cytosolic Ca^2+^ (reviewed in [16,146,147]). While not much is known about the role of DACCs in plants, it is assumed they cause shorter and transient Ca^2+^ influxes in response to various stimuli, such as cold stress and microbiotic stress [148,149]. The two most likely families of genes coding for HACCs, DACCs, and VICCs in plants are the glutamate-like receptor (GLR) and cyclic nucleotide-gated channel (CNGC) gene families, which consist of 20 members each in *Arabidopsis*. Other major Ca^2+^ permeable channels are mechanosensitive Ca^2+^-selective channels, MscS-like (MSL) and *mid1*-complementing activity (MCA) channels, Annexins, Two Pore Channels (TPCs), and Reduced hyperosmolarity-induced Calcium Increase channels (OSCAs) (reviewed in [147,150]).

Plant CNGCs have been found at the plasma membrane, the tonoplast and the endoplasmic reticulum, as well as restricted to the nuclear envelope [151,152]. They share structural similarity with their animal counterparts and are composed of tetrameric complexes that form non-selective cation channels permeable to several monovalent (e.g., K^+^ and Na^+^) and divalent (e.g., Ca^2+^, Ni^2+^, Sr^2+^, and Pb^2+^) cations [153,154]. However, their activation upon binding of cyclic nucleotides (cNMPs), such as cGMP and cAMP, remains controversial [152].

The plant glutamate-like receptors share structural and sequence similarities to the mammalian ionotropic glutamate receptors (iGluRs), which are involved in neurotransmission [155,156]. Like CNGCs, plant GLRs are non-selective cation channels that are thought to function as Ca^2+^ channels in several cellular processes in plants [121,122,126,157,158,159,160,161]. Based on extensive knowledge of the mammalian iGluRs [162,163], it seems that the domain structures, the overall membrane topology and channel orientation are partly conserved between plant GLRs and animal iGluRs [156,164,165,166,167,168]. However, important differences between animal iGluRs and plant GLRs can be detected in the primary sequence of several key domains, such as the pore region and the transmembrane domains that determine the channel selectivity [169]. Therefore, due to the poor conservation of the pore region between iGluRs and GLRs, it is unfortunately impossible to accurately determine the GLR selectivity based on what is known for iGluRs [170]. Indeed, thus far little is known about GLR selectivity in plants. However, it was recently shown that the moss channel PpGLR1 and the *Arabidopsis* channels AtGLR3.2 and AtGLR3.3 are permeable to cations, including Ca^2+^ [126,171]. Interestingly, while several GLRs, such as AtGLR1.4 and AtGLR3.4 have been shown to function as ligand-gated channels in heterologous systems [172], it seems that some GLRs are active without the need of a ligand [122,126,171]. GLRs have been shown to localise at the plasma membrane (e.g., [172,173,174,175]), the ER [176], in the chloroplasts and mitochondria [177,178], and in sperm cell (endo)membranes and the vacuolar membrane [171]. The tonoplast contains another important voltage-activated Ca^2+^-permeable channel. This channel was initially identified as a slow vacuolar (SV) channel that is activated by increases in cytosolic Ca^2+^ and membrane potential at the tonoplast [179,180]. The SV channel in Arabidopsis was later shown to be TPC1, a member of the conserved two-pore channel (TPC) subfamily of eukaryotic voltage- and ligand-gated cation channels [181]. Recently, the crystal structure of the vacuolar Arabidopsis TPC1 protein was reported [182,183] However, while TPC1 is permeable to Ca^2+^, it is also permeable to various monovalent and divalent cations, such as K^+^, Na^+^, and Ba^2+^ [184,185,186]. Therefore, it is thought that TPC1 is important for the regulation of cytosolic ion concentrations [187,188]. Importantly, under physiological conditions, TPC1 likely functions as a K^+^ channel rather than a Ca^2+^ channel [188]. These authors suggested that the observed Ca^2+^ changes in loss- and gain-of-function TPC1 lines are indirect, via another, unidentified Ca^2+^ channel in the tonoplast or via proton-coupled Ca^2+^ transport.

Mechanical stimuli, such as touch or wind, induce rapid and transient increases in cytosolic Ca^2+^ levels [15,189]. In plants, these mechanosensitive Ca^2+^ responses are thought to be mediated by two classes of putative mechanosensitive Ca^2+^-selective channels (MSCCs): MSL and MCA channels [3,190]. There are ten MSL genes in *Arabidopsis*, which code for homologs of the mechanosensitive channels of small (MscS) conductance in bacteria [191,192]. While the MSL proteins are prime candidates for being mechanosensitive Ca^2+^-permeable channels in plants, electrophysiological studies suggest that MSL proteins are primarily permeable to Cl^-^ rather than to Ca^2+^ [193,194]. Therefore, it remains unclear if they are involved in mechanical stimuli-induced Ca^2+^ signalling. Mid Complementing Activity1 (MCA1) was discovered in a functional complementation screen of an Arabidopsis cDNA library in a mutant of the *Saccharomyces cerevisiae* mechanosensitive Ca^2+^-permeable channel MID1, in which MCA1 could partially complement the conditional lethality of the *mid1* mutant [195]. Besides MCA1, Ca^2+^ uptake has also been shown for its only paralog in Arabidopsis, MCA2, and for homologs in rice (OsMCA1) and tobacco (NtMCA1 and NtMCA2) [196,197,198], but not for maize [199]. Additionally, electrophysiological experiments in *Xenopus* oocytes showed that MCA1 can act as a mechanosensitive channel, and that MCA2 is able to produce membrane stretch-activated currents [200]. Together, these observations suggest that the MCA proteins function as Ca^2+^-permeable mechanosensitive channels in plants.

Unlike conventional ion channels, Annexins are not exclusively membrane-bound or inserted, but are also found as soluble proteins in the cytosol and extracellular matrix [201]. They can form Ca^2+^-permeable channels across lipid bilayers [202,203] that contribute to cellular Ca^2+^ influx in plants [204,205]. Annexin-mediated Ca^2+^ transport seems to be regulated by several reactive oxygen species (ROS), such as hydroxyl radicals (OH^•^) and hydrogen peroxide (H_2_O_2_) [205,206,207]. Furthermore, it is hypothesized that Annexins may be involved in the transient elevations of [Ca^2+^]_cyt_ that are induced by extracellular ATP and ADP via their ATPase and GTPase activities [208,209].

Recently, hyperosmolality induced [Ca^2+^]_cyt_ increase 1 (OSCA1.1) and Calcium Permeable Stress-gated cation Channel1 (CSC1/OSCA1.2) were identified as hyperosmolality-gated Ca^2+^-permeable channels [210,211]. Both OSCA1 and CSC1 are non-selective cation channels, in which OSCA1 even had a slight preference for K^+^ over Ca^2+^ [211]. In Arabidopsis, OSCA1 belongs to a gene family with fifteen members, and homologues are present in other plant species and eukaryotes as well [212]. Both studied OSCAs localized to the plasma membrane, but a mutant in a the more distant OSCA4.1 shows vacuolar trafficking defects [213], suggesting a localisation in the late endosomal pathway.

## 3. Ca^2+^ Efflux Mechanisms

When a Ca^2+^ signalling event has been concluded by successfully inducing a cellular response, it is necessary that the [Ca^2+^]_cyt_ is restored to its resting levels. While Ca^2+^ channels are responsible for the fast influx of Ca^2+^ into the cytosol after detection of specific stimuli, the active efflux of Ca^2+^ out of the cytosol after a Ca^2+^ signalling event is regulated by three classes of Ca^2+^ transporters: P-type Ca^2+^-ATPases, Ca^2+^/H^+^ antiporters (CAX) and possibly cation/Ca^2+^ exchangers (CCXs).

Ca^2+^-ATPases are Ca^2+^ efflux pumps that are directly energized by ATP and belong to the second subclass of phosphorylated (P)-type ATPases (P_II_-ATPases), a family of ion pumps that are ubiquitous in all life forms. In plants, they can be further subdivided in two groups: P_IIA_- and P_IIB_-ATPases [214]. Arabidopsis encodes for four P_IIA_-ATPases or Endoplasmic Reticulum-type Ca^2+^-ATPases (ECAs) and ten P_IIB_-ATPases or autoinhibited Ca^2+^-ATPases (ACAs) that contain an autoinhibitory N-terminal region [214]. In addition to ECAs and ACAs, a P_I_-ATPase, HMA1, is a Ca^2+^/heavy metal transporter that localizes to the chloroplast envelope and is able to transport Ca^2+^ and heavy metal ions with high affinity [215,216]. The ACAs are activated upon binding of calmodulin to their regulatory domain [217] and their activity is further modulated by phosphorylation CBL-CIPK kinase complexes [218]. The ECAs and ACAs have a high-affinity for Ca^2+^ (K_m_ = 0.1–2 μM) but are low-capacity transporters that are mainly involved in maintaining the low resting cytosolic Ca^2+^ levels [219]. Indeed, the *aca8* mutant has higher resting Ca^2+^ levels in mature leaves, but has also higher peak responses to wounding and delayed recovery in response to extracellular ATP in seedling root tip cells [218].

The Ca^2+^/H^+^ antiporters, or cation exchangers (CAX), are high-capacity pumps with a relatively low affinity for Ca^2+^ (K_m_ = 10–15 μM) that drive the efflux of Ca^2+^ and some other divalent cations, such as Cd^2+^, against their concentration gradient by utilizing energy generated by the electrochemical proton gradient [220,221,222,223]. Therefore, it is assumed that the main role of these Ca^2+^/H^+^ antiporters is the initial reduction of the cytosolic Ca^2+^ concentration back to a few micromolar after a Ca^2+^ signalling event [219]. The CAX proteins seem to act in tandem to ACAs [224]. There are six known genes coding for CAX in Arabidopsis [225,226], five cation/Ca^2+^ exchanger (CCX) proteins and four more genes coding for EF hand-containing antiporters, making them potential Ca^2+^-selective antiporters involved in Ca^2+^ efflux [226]. CAX antiporters are mainly located on the tonoplast [221,227,228,229] and the plasma membrane [230,231] The rice CCX2 localizes to the tonoplast [232], while the Arabidopsis CCX2 localizes to the ER [233].

## 4. Ca^2+^ Sensing Mechanisms

Jointly, the stimuli-specific coordinated activation and inhibition of different influx and efflux systems shapes Ca^2+^ signatures that are further translated into corresponding molecular and biochemical cellular responses. For this purpose, plants have acquired an extensive set of Ca^2+^-binding proteins that can detect and convert Ca^2+^ signatures into a wide variety of biochemical responses. These so-called Ca^2+^ sensors can be classified into two main categories: sensor relays and sensor responders [234].

Sensor relays are Ca^2+^ sensors that contain a Ca^2+^ binding domain but lack other effector domains and are thus unable to transduce the Ca^2+^ signal by themselves. Therefore, they have to bind with specific interaction/s partner/s in order to transmit Ca^2+^ signatures. Two prominent groups of sensor relays in plants are the calmodulins (CaM) and calcineurin-B like (CBL) proteins, which are able to bind Ca^2+^ ions via four and three EF-hand domains, respectively [235]. The calcineurin-B like proteins interact with CBL-interacting protein kinases (CIPKs; [236]). Thus far, 10 CBLs and 26 CIPKs have been identified in Arabidopsis [237]. CBLs (Ca^2+^ binding function) and their corresponding CIPKs (kinase activity) can form flexible modular complexes that—depending on their composition—are partly responsible for the temporal and spatial specificity of Ca^2+^ signals in plant cells [238]. The CaM form a major class of Ca^2+^ sensors and are strongly conserved among eukaryotic organisms. They undergo large conformational changes upon binding to Ca^2+^ ions, which allows them to associate with a wide variety of interaction partners (such as transcription factors, protein kinases, protein phosphatases, receptors, metabolic enzymes, cytoskeleton-associated proteins, and ion channels and pumps [235,239,240,241,242,243,244,245,246]. In Arabidopsis, four CaM isoforms that share 97–99% sequence identity with each other have been identified, which are encoded by seven genes (AtCaM1–7; [242]). Furthermore, while these CaM isoforms (also called “typical CaM”) are strongly related to their counterparts in other eukaryotic organisms, plants possess several unique CaM-like proteins (CMLs; 50 in Arabidopsis) and downstream effector proteins not found in other eukaryotes [242]. These CMLs generally have at least 16% sequence identity with the typical CaMs and possess two to six EF-hand domains, without any other functional domains [247]. Interestingly, CMLs have been localised to different subcellular compartments, including mitochondria and peroxisome [248], nucleus [249], plasmodesmata [250], and endomembrane systems [251], suggesting specialized functions in these compartments.

In contrast to sensor relays, sensor responders are able to both bind Ca^2+^ and induce a response (e.g., protein kinase activity), without the need of interaction partners [234]. A prominent group of sensor responders in plants are the Ca^2+^-dependent protein kinases (CPKs), of which 34 members are present in Arabidopsis [252]. They are able to bind Ca^2+^ ions via four conserved EF hand domains in their C-terminal region, which leads to a conformational change and the subsequent activation of their kinase domain, thus allowing the phosphorylation and regulation of a downstream target [253,254,255]. Additionally, full activation of CPKs is achieved by autophosphorylation. CPKs are involved in various Ca^2+^ signalling-dependent biological processes, such as drought and salt stress (CPK10 and CPK11; [256]), stomatal closure (CPK3 and CPK6; [257]), ABA-responsiveness of guard cells (CPK4 and CPK11; [258]), regulation of ROS production [259], plant immunity [260] and cytoskeleton regulation [261].

### 4.1. Inhibition Strategy 1: Ca^2+^ Availability and Chelation

The most straightforward way to interfere with Ca^2+^ signalling is to modify the amount of available Ca^2+^. In the growth medium for in vitro plant tissue culture, CaCl_2_ is often included in millimolar concentrations, and can thus be adjusted by simply altering the recipe of the growth medium. The pectin in the plant cell wall can bind Ca^2+^ and can thus act as a reservoir for Ca^2+^ that can buffer periplasmic Ca^2+^ changes. Washing the apoplast with low Ca^2+^ medium probably only results in a moderate reduction of Ca^2+^. When performing such experiments, one should consider that intraorganellar communication can result in exchange of Ca^2+^ and thus concomitant reduction of the Ca^2+^ levels in intracellular Ca^2+^ stores [262]. However, the extent and dynamics of such intraorganellar Ca^2+^ exchange in plants is poorly characterized.

Another strategy to control the free Ca^2+^ levels is via Ca^2+^ chelators, such as ethylene glycol-bis(β-aminoethyl ether)-*N*,*N*,*N*′,*N*′-tetraacetic acid (EGTA), ethylenediaminetetraacetic acid (EDTA), and 1,2-bis(o-aminophenoxy)ethane-*N*,*N*,*N*′,*N*′-tetraacetic acid (BAPTA), which bind to Ca^2+^ ions with high affinity in a selective, reversible manner. Intracellular Mg^2+^ concentrations typically are 1000 to 10,000 times higher than the Ca^2+^ concentrations. Therefore, EGTA and BAPTA are much better Ca^2+^ chelators for biological systems due to their higher selectivity for Ca^2+^ over Mg^2+^ compared to EDTA, which interacts with a broader range of metal ions.

EGTA has high selectivity for Ca^2+^ over Mg^2+^ (3.8 × 10^5^ higher) and has a low K_d_ at neutral pH (K_d_ = 70–376 nM; [23]), allowing for Ca^2+^ buffering over a range of intracellular Ca^2+^ concentrations. BAPTA can bind two Ca^2+^ ions at once and is even more selective for Ca^2+^ than EGTA. Besides a slightly higher affinity for Ca^2+^ and a K_d_ between 110–220 nM over a wide pH range, BAPTA can bind and release Ca^2+^ ions about 50 times faster than EGTA (Ca^2+^ binding rate BAPTA = 4 × 10^8^ M^−1^ s^−1^ vs. EGTA = 1 × 10^7^ M^−1^ s^−1^; Ca^2+^ release rate BAPTA = 88 s^−1^ vs. EGTA = 0.7 s^−1^) [35]. These features explain why BAPTA has been a popular alternative to EGTA for controlling the level of both intracellular and extracellular Ca^2+^. While having a comparable K_d_, the difference in speed of Ca^2+^ binding between EGTA and BAPTA can be used to discriminate between nanodomain- and microdomain-coupling between Ca^2+^ sources and sensors (Reviewed in [23]). Therefore, they are often used to form Ca^2+^ buffers with well-defined Ca^2+^ concentrations or for controlling the Ca^2+^ concentration while studying the role of Ca^2+^ in cellular processes or in biochemical reactions.

Ca^2+^ chelators have been extensively used in the plant field for decades to explore the role of Ca^2+^ signalling in various physiological process. For instance, chelation of Ca^2+^ was able to suppress NH_3_-triggered Ca^2+^ response, [31], prevents PAMP-induced NO generation in tobacco cell cultures [28] and Arabidopsis leaf cells [24], inhibits floral induction of *Pharbitis nil* [26], inhibits Ca^2+^ transients that control stomatal aperture [27,34], and root gravitropism [29]. However, while EGTA and BAPTA have excellent Ca^2+^ chelator properties, they have some clear limitations that should be taken into account when planning an experiment. One property of Ca^2+^ chelators is that they are not only in equilibrium with Ca^2+^ ions but also with H^+^, in which Ca^2+^ binding to a chelator usually releases H^+^ (thus lowering the pH). The opposite also holds true for pH sensitive chelators such as EGTA, in which a change in pH can drastically reduce the Ca^2+^ buffering capacity [33]. Thus, it is important to use a pH buffer alongside the Ca^2+^ chelator to avoid undesirable effects of pH changes on its ability to buffer Ca^2+^ ions [30]. Importantly, while optimal EGTA-based Ca^2+^ chelation requires pH 8.0, most plant tissue culture media are often more acidic (pH 5.8). The Ca^2+^ association constant of BAPTA is relatively pH insensitive. Long-term Ca^2+^ depletion may lead to membrane destabilization and thus a large number of artefacts, such as increased permeability of the membranes. In addition, long EGTA treatments could modify the cell wall properties through reducing the levels of Ca^2+^ bound pectin. It is also important to realize that Ca^2+^ ions and their chelators are in equilibrium, which means that it is not possible to eliminate completely Ca^2+^, even by increasing the Ca^2+^ chelator concentrations to high levels [30]. Furthermore, the charges in BAPTA and EGTA prevent them from penetrating the cell membranes, and thus only lower the extracellular Ca^2+^. This can be overcome either by injection of the chelators into the cell [25] or by using acetoxymethyl ester forms of the chelators (BAPTA-AM, EGTA-AM) [38]. The acetoxymethyl ester protects the carboxylic groups, thereby neutralizing the charges of the chelators, allowing them to cross the cell membrane. When cytoplasmic esterases cleave the AM groups, the chelators are released and become trapped inside the cell. Importantly, in plants, AM groups can be cleaved in the apoplast due to the presence of extracellular esterases. To improve loading of the AM-esters, esterase inhibitors such as serine can be used [263]. An additional caution to be noted for Ca^2+^ chelators is that they can induce physiological effects independent of their Ca^2+^ chelating activity, such as modification of Cl^-^ channel activities and depolymerization of actin filaments and microtubules [32,36,37]. These observations of Ca^2+^ chelation-independent effects could complicate the interpretation of studies of Ca^2+^-regulated processes using EGTA and BAPTA.

### 4.2. Inhibition Strategy 2: Inhibition of Ca^2+^ Entry

Upon detection of an elicitor or perception of a mechanical or abiotic stimulus, increases in cytosolic free Ca^2+^ concentration occur by a tightly regulated influx of Ca^2+^ ions via specific Ca^2+^ channels in different membranes. The Ca^2+^ influx machinery is therefore a key objective to understand any Ca^2+^ based signalling cascade. However, in plants the most prominent putative Ca^2+^-permeable channels belong to large gene families [17,264]. The genetic complexity that is associated with functional redundancy has often discouraged plant Ca^2+^ biologists to determine the molecular nature of the Ca^2+^ channel in their process of interest. Therefore, an interesting alternative approach to manipulate Ca^2+^ channels is the use of inhibitors that target and block a range of Ca^2+^ channels. This approach is commonly used in the metazoan Ca^2+^ research and has led to an extensive set of reasonably selective Ca^2+^ channel inhibitors in these cell types.

## 5. Non-Selective Ca^2+^ Channel Blockers

The trivalent cations lanthanum (La^3+^) and gadolinium (Gd^3+^) are rare earth metals that non-selectively block cation channels [45,58,59] and stretch-activated Ca^2+^-permeable channels [60,61,62,63] in a wide variety of cell types of animals and plants [55]. They are bulkier than Ca^2+^ and hence physically block the pore of the Ca^2+^ channels.

While often disregarded, these trivalent cations do much more than just block Ca^2+^ channels. They bind with high affinity to most Ca^2+^-binding sites [47], and thus activate Ca^2+^ signalling, displace Ca^2+^ from membranes [40] or block Ca^2+^ efflux from the cell via interaction with Ca^2+^ ATPases [53,54]. In addition, besides blocking Ca^2+^ influx through Ca^2+^-permeable channels, La^3+^ and Gd^3+^ may have additional biological effects beyond their Ca^2+^ effects [44]. Indeed, there are indications that La^3+^ and Gd^3+^ have an effect on cell proliferation [48,52,57] and apoptosis [42,46]. Furthermore, La^3+^ and Gd^3+^ can form insoluble salts in the presence of low phosphate concentrations [43], a feature that is used to remove and recover phosphate from waste water [56] and that has important implications for working in plants. Consistently, at concentrations greater than 100 µM, La^3+^ and Gd^3+^ cause root architecture changes that can be explained by a phosphate starvation response [50]. Moreover, rather than inhibiting Ca^2+^ signalling, La^3+^ and Gd^3+^ treatments can even trigger prominent Ca^2+^ signals similarly as Al^3+^ [49], via an unknown mechanism. Such a La^3+^ induced response can be avoided when phosphate is omitted from the medium [51]. Similarly, while La^3+^ blocks the calcium influx in the short-term response to low K^+^, 25 μM La^3+^ enhances the long-term K^+^ deprivation-induced secondary Ca^2+^ signal in Arabidopsis roots [39]. Moreover, one should take into consideration that the activity of La^3+^ and Gd^3+^ may depend on the Ca^2+^ concentration in the medium [41]. Despite these important side-effects, La^3+^ and Gd^3+^ remain to be extensively used to study Ca^2+^ signalling in both the plant and animal fields. 

Another compound that has been used for decades as a non-specific Ca^2+^ channel blocker is Ruthenium Red (RR). RR is a synthetic crystalline inorganic polycationic dye that strongly binds to phospholipids, fatty acids, and mucopolysaccharides, thus explaining its original use as a tissue dye for electron microscopy [81]. Besides its use as a dye, RR was also initially shown to inhibit mitochondrial Ca^2+^ transport [72] and Ca^2+^ release from the sarcoplasmic reticulum in animals [75,76]. Later, it was demonstrated that RR was also able to inhibit various Ca^2+^-permeable channels and Ca^2+^-binding proteins, both in animals and plants [64,67,68,69,70,71,73,77,79]. Furthermore, RR is a potent inhibitor of SV channel currents, in which at least two RR ions binds deep within the channel pore in a cooperative way [74]. RR could inhibit NaCl-induced Ca^2+^ wave propagation, which was proposed to be mediated by TPC1 [79]. However, it remains unclear if this reflects a direct effect of RR on TPC1 activity. More recently, it was demonstrated that RR interacts with the C-terminal region of Piezo channels in animals, and is able to block the inward currents through these channels [65,66]. The effects of RR on Ca^2+^ are highly pleiotropic as it can also Inhibits the mitochondrial Ca^2+^ uniporter, Ca^2+^-ATPases, troponin C, and CaM [74,78,80,82].

## 6. L-Type Calcium Channel Antagonists

The largest and best-characterized group of Ca^2+^ channel blockers in animals are antagonists of L-type voltage-dependent Ca^2+^ channels (VDCCs) (reviewed in [90]). These Ca^2+^ channel blockers have been used for decades in the treatment of hypertension, cardiac ischemia, arrhythmias, and angina [90,112], and are some of the most widely prescribed drugs in the world. Furthermore, these drugs have helped unravel the physiological role of various L-type Ca^2+^ channels in animal field Ca^2+^ research [97,99,113]. The main L-type Ca^2+^ channel blockers that are used in plants are dihydropyridines (DHP, e.g., nifedipine), phenylalkylamines (PAA, e.g., verapamil) and bepridil, each with different receptor sites, selectivity, and specificity [90,94].

While L-type VDCCs, which are targeted by these Ca^2+^ channel blockers in animals, do not exist in plants, plants do have binding sites for L-type VDCC blockers. Binding to plant membranes was also demonstrated biochemically for verapamil and dihydropyridine derivatives [98,104,105,115]. Binding sites of L-type VDCC blockers can be visualized as fluorescently-tagged dihydropyridines and phenylalkylamines (e.g., DM-bodipy-PAA and DM-bodipy-DHP) that decorate several membranous structures in the plant cell [102,103,110,116]. Furthermore, DM-bodipy-PAA binding can be competed out with non-fluorescent bepridil and verapamil [110,111], suggesting that bepridil and verapamil can bind to the same targets in plant membranes.

However, the molecular nature of these inhibitor binding sites in plants remains elusive and electrophysiological data supports that these inhibitors interfere with Ca^2+^ currents in plants. Verapamil blocks Ca^2+^ uptake in rice roots [106], and blocks the inward Ca^2+^ current of the *rca* channel, a Ca^2+^-selective channel in the wheat root plasma membrane, in a concentration- and voltage-dependent manner [107,108]. In tomato, HACC currents could be partially inhibited by nifedipine, with a half-blocking concentration of 100 µM [83]. Associated with these effects on Ca^2+^ fluxes, L-type Ca^2+^ channel blockers disrupt a number of Ca^2+^ regulated processes in plants. Recently, it was shown that the Ca^2+^ influx in the root apex induced by low copper concentrations is inhibited by verapamil [109]. Tip growth of root hairs could be stopped by nifedipine, indicating that Ca^2+^ influx through Ca^2+^-permeable channels is necessary for normal root hair tip growth [84]. In pollen tubes, nifedipine inhibits the grain in vitro germination [87] and alters the Ca^2+^ tip gradient and growth in Lily tubes [89]. Cytokinin-induced budding in the moss *Funaria* was largely inhibited by nifedipine, while photoreversed inactive nifedipine had no significant effect on budding [85]. Interestingly, the instant photoconversion of nifedipine into an inactive form by ultraviolet light can be used to quickly inactivate nifedipine and thus restore Ca^2+^ currents [86,88,91].

While L-type Ca^2+^ channel blockers have been used efficiently to block voltage-activated Ca^2+^ channels in plant, it must be noted that L-type Ca^2+^ channel blockers are known to interact with animal Na^+^ and K^+^ channels [92,93,95,96,265], and verapamil, nifedipine, and bepridil have been shown to directly inhibit outward rectifying K^+^ channels (ORKs) in plants [100,101]. This calls for caution when using L-type Ca^2+^ channel blockers to show the involvement of Ca^2+^ channels in plant physiological processes, since the observed effects of these inhibitors could be the result of inhibition of K^+^-influx, rather than Ca^2+^-influx related. On the other hand, these findings hint at common structural elements between plant outward rectifying K^+^ channels and animal voltage-dependent Ca^2+^ channels, suggesting that they may have evolved from a common ancestral gene [266]. Recent crystal structural data has shown that the TPC1 and CNGCs channels of plants look similar to ORKs, further suggesting that these types of putative Ca^2+^-permeable channels could be direct targets of L-type Ca^2+^ channel blockers in plants [182,183,267]. A photoaffinity labeled azido derivative allowed to purify peptide, which forms a non-selective Ca^2+^ channel when incorporated in giant liposomes [268,269]. Interestingly, the size of this peptide was about 75 kDa, which corresponds to the size of CNGCs. In addition, verapamil could inhibit OsTPC1 mediated Ca^2+^ flux [114]. The crystal structure of TPC1 showed that the pharmacophore *trans*-NED19, which prevents infection by Ebola virus and Filoviruses in animals, could bind and inhibit ion conductance of TPC1 [183,270]. Similarly, verapamil and the dihydropyridine nimodipine also inhibit Ebola virus infection [270], corroborating that verapamil targets plant TPCs.

Another piece of caution is that L-type VGCC inhibitors such as bepridil can directly inhibit CaM, similarly to trifluoperazine [117,118]. Therefore, additional the observed effects could be caused by interfering with CaM-regulated proteins, rather than with Ca^2+^-permeable channels.

## 7. iGluR/GLR Antagonists

As was discussed earlier, the *GLR* genes form a large gene family of putative ligand-gated Ca^2+^ channels in plants that possess structural and sequence similarity to the mammalian ionotropic glutamate receptors. Several established iGluR agonists and antagonists are also used to target plant GLRs, with 6,7-dinitroquinoxaline-2,3-dione (DNQX) and 2-amino-5-phosphonopentanoic acid (AP5) being the most commonly used in plants. DNQX is thought to function on plant GLRs by attaching inside their ligand-binding sites, while AP5 is probably binding to their l-glutamate binding site, thus competing with the natural ligand [119]. Similar to DNQX, 6-cyano-7-nitroquinoxaline-2,3-dione (CNQX) and 5,7-dinitro-1,4-dihydro-2,3-quinoxalinedione (MNQX) are also often used to inhibit iGluRs and plant GLRs [124,271]. However, for most iGluR agonists and antagonists it has yet to be demonstrated whether they are active in plants by directly binding to plant GLRs.

Several popular competitive antagonists of iGluRs, namely DNQX, CNQX, and AP5, were able to inhibit pollen tube growth in tobacco seedlings [122]. In *Arabidopsis* papilla cell protoplasts, the same inhibitors were also shown to inhibit the [Ca^2+^]_cyt_ increases triggered by the pollen ligand SP11/SCR (S-locus protein 11) [120]. Furthermore, the Ca^2+^ currents that are induced by microbe-induced molecular patterns (MAMPs) were blocked by several animal iGluR antagonists [121]. Besides these examples, DNQX, CNQX, MNQX, and AP5 have been used by several groups to study GLR function in whole plants and organelles (e.g., [119,123,124,125]). Also in the moss *Physcomitrella patens*, PpGLR1 currents could be inhibited by CNQX and AP5 [126]. The hypothesis that DNQX acts by directly binding to plant GLRs was further supported by a molecular modelling approach, where it was shown that DNQX is able to bind into the predicted ligand-binding pocket of AtGLR2.9 [119]. This observation is consistent with its proposed mode of action in which DNQX prevents binding of GLR agonists (such as glutamate and glycine) to GLRs by blocking their ligand-binding pocket, based on the predicted interaction of DNQX with animal iGluRs. Importantly, DNQX could inhibit AtGLR3.4-mediated Ca^2+^ currents in planar lipid bilayers, corroborating a direct effect on GLR-function [123]. Therefore, it is probably safe to assume that the effects of DNQX and its related iGluR antagonists CNQX and MNQX (5,7-Dinitro-1,4-dihydro-2,3-quinoxalinedione) on Ca^2+^ signals reflect the inhibition of GLR3.4 and related GLRs [123,124].

## 8. Inhibition Strategy 4: Inhibition of CaM-Based Ca^2+^ Signalling

A popular inhibitor target is CaM, the most important intracellular Ca^2+^-binding protein in eukaryotic organisms. The high degree of sequence conservation makes that many of these CaM antagonists are also active in plants. They can interfere with canonical CaMs, probably several CaM-likes and CPKs, which have a CaM-based auto-inhibitory domain.

The oldest and most commonly used CaM antagonists include the phenothiazines (e.g., fluphenazine, trifluoperazine (TFP) and chlorpromazine), which strongly interact with CaM in a Ca^2+^-dependent manner and block its ability to activate enzymes [128,130]. These drugs possess structural features that are also present in multiple CaM binding peptides and proteins, suggesting that they likely function as competitive inhibitors for CaM binding [272,273,274]. Besides the phenothiazines, the sulfonamide W-7, the imidazole calmidazolium and a broad range of structurally diverse, natural products derived from plants, bacteria, and fungi have been shown to also inhibit CaM to varying degrees, either by interacting with CaM directly or with a CaM-containing enzyme complex in a competitive or non-competitive way. These compounds mostly consist of alkaloids and peptides, but multiple other natural CaM antagonists have been identified, including multiple polyamines, terpenoids, anthracyclins and anthraquinones, lignans, xanthones, stilbenoids, polyketides, and some flavonoids, and other phenolic compounds (reviewed in [275,276]). To date, some of the most potent CaM antagonists consist of some toxic peptides derived from animal venoms (e.g., melittin, apamin, and mastoparans; [277,278]) and polymyxin, a cyclic peptide antibiotic [279], making them interesting tools to further explore CaM-mediated signalling pathways. The phytotoxic effect of several CaM antagonists is thought to be caused by their CaM-inhibitory activity, as is the case for the phytotoxin ophiobolin A, which binds to specific Lys-residues in CaM [133].

Besides several CaM isoforms, higher plants also contain calmodulin-like (CML) proteins and Ca^2+^ dependent protein kinases (CPKs) in which the autoinhibitory domain consists of a CaM moiety. Via such a vast array of potential targets, CaM antagonists elicit strong pleiotropic effects in plants. This ranges from secretion [280], mitotic progression [281], auxin transport [282], gravitropism [283], red and blue light-induced acidification by leaf epidermal cells [284], inhibition of cytokinin-induced bud formation in the moss *Funaria* [285], plant growth and defense [239], and peroxisomal Ca^2+^ and protein import [286,287]. Additionally, several CaM inhibitors (W-7, TFP and calmidazolium) induce a cytosolic calcium increase [127,129] which itself could be the cause of some of the effects observed after CaM antagonist treatment, rather than a direct effect of CaM inhibition. The pleiotropic effects of CaM inhibitors preclude their use in long-term treatments, but do not preclude their use to dissect CaM-regulated biochemical activities, such as CPK activity [131,132,288,289,290,291]. As a control in experiments with W-7, one often uses the inactive structural analog N-(6-aminohexyl)-1-naphthalenesulfonamide (W-5) [24].

### 8.1. Activation Strategy 1: Facilitated Ca^2+^ Transport across Membranes

Instead of inhibiting Ca^2+^ influx mechanisms, it is sometimes desirable to induce or facilitate the influx of Ca^2+^ ions across membranes. For this purpose, several methods are available. One approach is the use of Ca^2+^ ionophores that directly facilitate the transport of Ca^2+^ across the plasma membrane. Two commonly used Ca^2+^ ionophores are A23187 (or calcimycin) and ionomycin.

A23187 is an antibiotic compound that also acts as an ionophore for divalent cations. While A23187 is most selective for Mn^2+^, it also functions as an efficient Ca^2+^ ionophore and has thus been used extensively to increase intracellular Ca^2+^ concentrations in intact cells ([134,136]). However, A23187 also acts as an uncoupler of oxidative phosphorylation and inhibits mitochondrial ATPase activity, causing pleitropic effects [135]. Furthermore, A23187 is intrinsic fluorescent and excitable by UV-light, making it not very compatible with some commonly-used UV-excitable Ca^2+^-indicators, such as Fura-2. In this case, 4-Bromo A23187, a non-fluorescent halogenated analogue of A23187, forms a good alternative [137]. In Arabidopsis treatment of root hairs with A23187 induced a steep rise in cytosolic [Ca^2+^]_cyt_ with a consequent strong reduction in the growth rate [15].

Ionomycin is another potent and highly selective Ca^2+^ ionophore. It is often used in studies of Ca^2+^ transport across biological membranes and to modify intracellular Ca^2+^ concentrations when studying cellular Ca^2+^ signalling processes. It is also used for the calibration of fluorescent Ca^2+^ indicators in animal cells [138]. As an alternative to the chemical Ca^2+^ buffers, one could also employ Ca^2+^ binding proteins, such as parvalbumin to locally buffer cytoplasmic Ca^2+^ signals [292]. In non-plant systems, optogenetic modulation of light-gated cation channels allows for very precise modulation of Ca^2+^ entry [293]. Yet, the currently used wavelengths overlap with the spectrum needed for normal plant growth, suggesting that plant growth under normal illumination may not be possible.

### 8.2. Activation Strategy 2: Inhibition of Ca^2+^ Efflux Machinery

All Ca^2+^ signals in the cytoplasm are dissipated via active efflux. Therefore, inhibition of the Ca^2+^ efflux machinery causes a passive increase in the cytoplasmic Ca^2+^. The most drugable components of Ca^2+^ efflux are ECA-type and ACA-type Ca^2+^ ATPases: Fluorescein derivatives, such as Erythrosin B and Eosin Yellowish (Eosin Y) are potent inhibitors of ACAs, [4,139,141], while ECAs are specifically inhibited by cyclopiazonic acid (CPA; [4,142]). Thapsigargin potently inhibits the Ca^2+^/Heavy metal pump AtHMA1 [215], but not ECAs [142], suggesting that thapsigargin only has minute direct effects on Ca^2+^ homeostasis. Eosin Y affects ACA activity with 10,000 fold greater affinity than that of H^+^ ATPases [139]. Therefore, at low working concentrations (0.2–0.5 µM; [4,140]), Eosin Y is likely to be relatively selective towards ACAs. However, the intrinsic fluorescence of fluorescein-derived ACA inhibitors limits their use in sensitive fluorescence based analyses. It should be noted that ACA activity also generates a counter current of H^+^ [294]. This implies that inhibition of Ca^2+^ ATPases may affect the intracellular pH. However, this proton current is independent of the concentration gradient [294], implying that Ca^2+^ ATPase activity cannot be modulated via the extracellular pH.

## 9. Conclusions and Perspectives

Plant Ca^2+^ signalling research has seen great progress over the last years. However, while great insights and understanding have been gained about the Ca^2+^ signalling components responsible for decoding and translating Ca^2+^ signatures, such as calmodulins, CMLs, CPKs, and CBL/CIPK complexes, our knowledge about the machinery responsible for generating these Ca^2+^ signatures severely lags behind. Many putative Ca^2+^-permeable channels have been identified in plants, including CNGCs, GLRs, Annexins, MSCCs, OSCAs, and TPC1, our knowledge of the physiological roles of these channels in Ca^2+^ signalling is still limited. While Ca^2+^ signalling mutants form promising tools for identifying more Ca^2+^-permeable channels in plants, this strategy is not without its drawbacks. Indeed, Ca^2+^-permeable channel families in plants often consist of many different structurally similar members, which can share significant functional redundancy between each other, thus hindering genetic studies on these channels. Therefore, the use of agonists and antagonists that specifically interact with Ca^2+^-permeable channels or other Ca^2+^-components forms an interesting alternative for studying Ca^2+^-signalling processes in plants. The currently most frequently used Ca^2+^ signalling blockers, such as L-type Ca^2+^ channel blockers, GLR antagonists and CaM antagonists, are mostly derived from the animal field. However, since the Ca^2+^ signalling machinery differs significantly between the plant and animal kingdoms, these inhibitors often have undesirable off-target effects or unknown targets *in planta*. Therefore, an obvious strategy to obtain specific Ca^2+^ channel inhibitors is to start an unbiased *de novo* chemical screen for inhibitors directly in plant systems. A suppressor screen of *cpr22* (AtCNGC11/12)-induced seedling lethality yielded 13 chemicals that partially restored seedling growth [295], which includes three putative Ca^2+^ channel blockers, dibucaine erythrosine B and diethylstilbestrol. However, it was not tested if these inhibitors affected the Ca^2+^ channelling activity of the hyperactive CNGC-based CPR22 channel [295]. Another approach may be to develop variants of L-type VGGC inhibitors that are more selective in plants. An essential task that lies in all these efforts is the identification of the molecular targets, and the electrophysiological characterisation of their activity. The increasing availability of detailed crystal structures of Ca^2+^-permeable channels, in combination with chemical compound screen approaches could lead to the identification of such plant specific Ca^2+^-signalling inhibitors, leading to the generation of a more robust toolset for studying plant Ca^2+^-signalling pathways in the future.

## Figures and Tables

**Table 1:** Overview of commonly used types of Ca^2+^ signalling antagonists and agonists in plants.

Ca^2+^ (ant)Agonist Type	Compound	Putative Target(s)	References
Ca^2+^ chelators	EGTA	Ca^2+^ ions >>> Mg^2+^ ions	[23,24,25,26,27,28,29,30,31,32,33,34]
	BAPTA	Ca^2+^ ions >>> Mg^2+^ ions	[23,25,27,32,34,35,36,37,38]
Non-selective Ca^2+^ channel blockers	Lanthanum (La^3+^)	cation channels, stretch-activated Ca^2+^-permeable channels, Ca^2+^ ATPases, most Ca^2+^-binding sites	[39,40,41,42,43,44,45,46,47,48,49,50,51,52,53,54,55,56]
	Gadolinium (Gd^3+^)	cation channels, stretch-activated Ca^2+^-permeable channels, Ca^2+^ ATPases, most Ca^2+^-binding sites	[43,45,47,49,50,55,57,58,59,60,61,62,63]
	Ruthenium Red	various Ca^2+^-permeable channels and Ca^2+^-binding proteins, MCU, SV channel, Ca^2+^-ATPases, CaM, Piezo	[64,65,66,67,68,69,70,71,72,73,74,75,76,77,78,79,80,81,82]
L-type calcium channel antagonists	Dihydropyridines	voltage-activated Ca^2+^ channels, HACC currents, ORKs	[83,84,85,86,87,88,89,90,91,92,93,94,95,96,97,98,99,100,101,102,103]
	Phenylalkylamines	voltage-activated Ca^2+^ channels, *rca* channel, ORKs, TPC1	[90,93,94,99,100,101,102,103,104,105,106,107,108,109,110,111,112,113,114,115,116]
	Bepridil	voltage-activated Ca^2+^ channels, ORKs, CaM	[100,101,103,110,111,117,118]
iGluR/GLR agonists and antagonists	DNQX	iGluRs and GLRs	[119,120,121,122,123,124,125]
	CNQX	iGluRs and GLRs	
	MNQX	iGluRs and GLRs	[124]
	AP5	iGluRs and GLRs	[120,121,122,125,126]
CaM antagonists	Phenothiazines	CaMs, CMLs	[127,128,129,130]
	W-7	CaMs, CMLs	[24,127,129,131]
	Calmidazolium	CaMs, CMLs	[129,131,132]
	Ophiobolin A	CaMs, CMLs	[133]
Ca^2+^ ionophores	A23187	Ca^2+^ ions	[15,134,135,136]
	4-Bromo A23187	Ca^2+^ ions	[137]
	Ionomycin	Ca^2+^ ions	[138]
P-type Ca^2+^-ATPase antagonists	Erythrosin B	ACAs	[4,139]
	Eosin Y	ACAs	[4,139,140,141]
	CPA	ECAs	[4,142]

EDTA, ethylenediaminetetraacetic acid; EGTA, ethylene glycol-bis(β-aminoethyl ether)-*N*,*N*,*N*′,*N*′-tetraacetic acid; BAPTA, 1,2-bis(*o*-aminophenoxy)ethane-*N*,*N*,*N*′,*N*′-tetraacetic acid; MCU, mitochondrial calcium uniporter; SV, slow vacuolar; CaM, Calmodulin; HACC, hyperpolarization-activated Ca^2+^-permeable channel; CAX, cation exchanger; iGluR, ionotropic glutamate receptor; GLR, glutamate receptor-like; DNQX, 6,7-dinitroquinoxaline-2,3-dione; CNQX, 6-cyano-7-nitro-quinoxaline-2,3-dione; MNQX, 5,7-Dinitro-1,4-dihydro-2,3-quinoxalinedione; AP5, 2-amino-5-phosphonopentanoic acid; CML, CaM-like; CPK, Ca^2+^ dependent protein kinase ; ACA, autoinhibited Ca^2+^-ATPase; ECA, Endoplasmic Reticulum-type Ca^2+^-ATPase; CPA, cyclopiazonic acid; ORK, outward rectifying K^+^ channel.
